# Effect of He^2+^ ion irradiation on the mechanical properties of automated fibre placement (AFP) CF-PEEK thermoplastics composites

**DOI:** 10.1038/s41598-023-45742-8

**Published:** 2023-11-01

**Authors:** Ebrahim Oromiehie, Vishnu Nair, Ken Short, Tao Wei, Dhriti Bhattacharyya, B. Gangadhara Prusty

**Affiliations:** 1 ARC Training Centre for Automated Manufacture of Advanced Composites, School of Mechanical and Manufacturing Engineering, UNSW Sydney, Sydney, NSW 2052 Australia; 2 School of Mechanical and Manufacturing Engineering, UNSW Sydney, Sydney, NSW 2052 Australia; 3 Australian Nuclear Science and Technology Organization (ANSTO), Lucas Heights, NSW 2234 Australia; 4 School of Materials Science and Engineering, UNSW Sydney, Sydney, NSW 2052 Australia; 5 Sovereign Manufacturing Automation for Composites CRC Ltd (SOMAC CRC), Sydney, NSW 2052, Australia

**Keywords:** Composites, Characterization and analytical techniques

## Abstract

Carbon fibre-reinforced polyetheretherketone (CF-PEEK) composites have gained significant usage across diverse industries like automotive and aerospace due to their desirable characteristics. These properties encompass recyclability, low density, high strength, wear resistance and thermal stability. The components made from CF-PEEK composites for space applications will be subjected to a high radiation environment due to the incoming cosmic rays, comprising protons, α particles, electrons, γ rays, etc., once they escape the Earth’s atmosphere. The ion irradiation of CF-PEEK is accompanied by radiation-induced effects, which drastically change the structure and properties of irradiated material. Since the resistance of CF-PEEK to radiation damage has not been studied extensively, this study aims to understand the effect of high-energy He^2+^ ions on the microstructure and properties of CF-PEEK composites manufactured using automated fibre placement (AFP) under different processing conditions. The samples have been radiated with 5 MeV He^2+^ ions using an energy degrader wheel to create a layer with relatively uniform damage. Then, were characterized using optical and scanning electron microscopy and their hardness was evaluated using nanoindentation. It was observed that, irradiation increases the hardness of the fibres in all cases. Also, fibre orientation affects the hardness in a statistically significant manner in both unirradiated and irradiated conditions.

## Introduction

There is a growing demand for the application of carbon fibre-reinforced polymer (CFRP) composites in aerospace and aviation industries, owing to their outstanding mechanical properties compared to conventional metals^1^. Among them, thermoplastic CFRP composites, such as carbon fibre-poly-ether-ether-ketone (CF-PEEK), are superior due to their excellent mechanical properties such as strength and fatigue resistance^2^, chemical inactivity and corrosion resistance^3,4^, recyclability, thermal stability and insulation characteristics, and wear resistivity^5,6^. These attributes enhance product performance, safety, and efficiency, aligning with the stringent requirements of these dynamic sectors.

While in service inside aircraft or spacecraft, these materials would be subjected to different types of radiation consisting of both high energy electromagnetic rays, such as γ rays and X-rays, and subatomic particles (cosmic rays). The latter include about 90% protons (H^+^), ~ 9% α (He^2+^) particles, ~ 1% β (e^−^) particles and a very small fraction of heavier nuclei^7^ that may cause damage as they penetrate through the material, by displacing the target atoms in their path from their original positions, leaving vacancies and interstitials. These can subsequently form voids and interstitial clusters, resulting in changes in microstructure and mechanical properties. Since the planned future space explorations and ongoing missions like the International Space Station and unmanned interplanetary voyages have to endure the harsh radiation environment of space for long durations, they will be subject to high fluences of ion irradiation over their lifetime. Thus, the behaviour of CF-PEEK composites under extreme conditions of long-term irradiation combined with thermal shock needs to be investigated^8^.

Recent investigations have shown that irradiation can change the structure of the fibres and resin system, including atomic displacements, defect formation, cross-linking, chain scission, surface modifications, thermal and oxidative effects, and crystal structure alterations, consequently changing their physical and chemical properties^9^. For instance, N^+^ ion irradiation resulted in diameter change and morphology of the carbon fibre due to ion sputtering, ion implantation and chemical reaction between CF and nitrogen atoms in the fluence range 10^17^–10^19^ cm^−2^^9^.

Numerous experimental studies have shown that the mechanical property changes in materials irradiated with high energy ions can be reliably measured using small-scale testing. The effect of ion irradiation on the tensile properties of pure Ni single crystals was investigated using an in-situ micromechanical testing device inside an SEM. The results showed that the peak strength increases from ~ 230 MPa for the unirradiated material to ~ 370 MPa and ~ 500 MPa for the materials irradiated with 6 MeV He^2+^ ions to peak damage of 10 displacements per atom (dpa) and 19 dpa, respectively^10^.

Effects of ion irradiation on microstructures and mechanical properties of SiOC nanocomposites containing β-SiC nanocrystals and turbostratic graphite were investigated under a series of radiation conditions using 4 MeV Kr ions, with the maximum fluences of 1 × 10^14^, 2 × 10^14^, 1 × 10^15^ ions/cm^2^. It was shown that ion irradiation results in the rearrangement of the Si-containing tetrahedral units with formation of more mixed-bonded units in the amorphous matrix, and disordering of the turbostratic graphite, but the crystallinity of the β-SiC is preserved. More severe radiation conditions result in higher variations in the amorphous SiOC matrix and greater damage to the graphite. However, the surface morphologies and mechanical properties showed little change under the applied radiation conditions, indicating the macro-property stability of the materials^11^.

The radiation damage and thermal shock response of carbon–carbon (C/C) composites and carbon fibre reinforced molybdenum-graphite compounds (MoGRCF) to long-term irradiation with intense proton beams (120–200 MeV) were investigated by Simos et al.^12^. The E-951 BNL experimental results (using 24-GeV proton tight beam pulses) confirmed that the C–C composite can withstand thermal shock better than graphite. The study revealed the similarity between the effects of fast neutrons and energetic protons on the microstructure of C/C and graphite composites around the threshold fluence of ~ 5 × 10^20^ cm^−2^. It also showed that modest irradiation levels of ∼6 × 10^18^ p/cm^2^ can induce significant changes in the microstructure of MoGRCF. The impedance properties of MoGRCF tested in the Large Hadron Collider (LHC) beam collimation exhibited a significant decrease in post-irradiation load–displacement behaviour even after low dose levels (∼5 × 10^18^ p/cm^2^).

The influence of Si and O ions, with 25 MeV energy, on the chemical and structural properties of PEEK films was investigated in the work by Yang et al.^13^. The results showed that the surface roughness of the PEEK material decreases after irradiation by both Si and O ions. Thermal properties of PEEK irradiated by 25 MeV O ions indicated a new secondary crystallization peak during the cooling stage. Accordingly, the low fluence irradiation increased the viscoelasticity and mechanical properties of PEEK films, while high fluence irradiation caused it to decrease.

It is well known that the characteristics of the impinging particles, including type and initial energy are very important determinants of the nature and extent of damage. For instance, electrons, ions and neutrons with the same initial energy result in vastly different microstructural effects. Since electrons are much smaller than ions, they carry much less momentum, and their ability to transfer energy and displace atoms from their positions in the target is much less compared to the heavier ions. For any given initial energy of the impinging particle, the fraction of recoil target atoms having a higher energy range is greater for ions compared to electrons^14^. In general, electrons produce a greater number of isolated point defects, while ions cause the formation of nanometre-scale defect clusters which are less mobile^15^. Thus, the size and spatial distribution of defects caused by electron and ion irradiation are different, and consequently, the resulting changes in mechanical properties are substantially divergent.

In a study relevant in this context, the changes in thermal properties induced by irradiation of 10 MeV H^+^, 20 MeV He^2+^ and 2 MeV electrons were studied for non-crystalline and crystalline PEEK using differential scanning calorimetry (DSC). The results revealed that cross-linking proceeds by irradiation in all cases studied. However, the probability of cross-linking in ion irradiation is substantially higher compared with that in electron irradiation. Isothermal crystallization also showed that the ion-induced damage is more severe than electron damage. In addition, the tensile properties for non-crystalline PEEK vary according to the difference in thermal properties between ion and electron irradiations. The difference between ion and electron irradiations was scarcely observed for crystalline PEEK^16^.

The effects of high-dose electron-beam irradiation on the mechanical properties of CF-PEEK composite, manufactured using compression moulding were studied in Sasuga et al.^17^. In this study, the irradiation was conducted in the air using 2 MeV electron beam from Dynamitron IEA-300-25-2 accelerator (dose rate 5kGysec-1). To prevent a temperature rise of the specimens during irradiation, they were wrapped using 20 µm thick aluminium foil. The results revealed that the mechanical properties at room temperature were less affected by irradiation up to 180 MGy. However, at 77 K the strength of irradiated samples (> 100 MGy) was slightly decreased. Also, at high temperatures (e.g. 413 K), the mechanical properties of irradiated specimens improved in contrast with unirradiated specimens. Moreover, the viscoelastic measurement showed an improvement in mechanical properties at high temperatures. This resulted from the high-temperature shift of the glass transition of the matrix PEEK and the formation of cross-linking in PEEK.

The evaluation of the mechanical properties of materials before and after irradiation can be conducted using nanoindentation testing^9^. The relationship between the ion energy, damage peak depth and hardness peak depth was investigated through a nanoindentation test on ion-irradiated SS316 samples (using He^2+^ ions having 1, 2 and 3 MeV beam energies)^18^. From the obtained radiation-induced hardness (ΔH) curves, it was observed that the peaks of the hardness curves are at a much shallower depth than those of the displacement damage curves, and the hardness peaks appear at increasing depths with increasing ion energies. These effects are caused by the large size of the plastic zone in ductile metals, and the increasing penetration of ions at higher energies, respectively.

Nanoindentation and nano-scratching responses of PEEK-based composites reinforced with short carbon fibre (CF) and nano-silica have been investigated and show a significant increase in hardness and elastic modulus due to the presence of reinforcing agents. The incorporation of short carbon fibres (6 mm) and 2 wt.% of nano-SiO_2_ particles into pure PEEK and conventional composite (PC) have resulted in 143% and 44% improvement in the reduced elastic modulus^5^.

In another study, the atomic force nanoindentation technique was used to investigate the effect of strain rate on the PEEK. It was shown that the average hardness and elastic modulus of PEEK follow a linear model (from 263.9 MPa/1.377 GPa to 323.1 MPa/2.477 GPa ) with respect to logarithm of strain rate from (0.012 s^−1^ to 1 s^−1^)^19^. This technique was also used to investigate the microscopic and mechanical properties of pure PEEK versus CF-PEEK. It was shown that the load–displacement curves of pure PEEK have superior uniformity, repeatability and consistency at different nano-indentation depths compared to CF-PEEK^6^.

Automated Fiber Placement (AFP) is one of the advanced manufacturing techniques that is being extensively used in the aerospace industry for manufacturing high-performance composite structures^20–22^. In-situ consolidation in AFP technology is performed through merging several manufacturing stages like cutting, curing and consolidation. Nevertheless, the quality and integrity of AFP-manufactured composites is heavily dependent on a large number of variables and parameters. Some of the key parameters in AFP are lay-up speed, curing temperature and consolidation force. The influence of AFP processing parameters on the microstructure and properties of CF-PEEK composites have been investigated in the authors’ earlier studies^23–28^.

The recent study on the nanomechanical characterisation of unidirectional CF-PEEK composites manufactured using automated fibre placement (AFP) showed that the nano hardness, elastic modulus, and creep resistance either in the polymer matrix or fibre/matrix interface of the samples that were manufactured at higher processing temperature are relatively higher than those which are manufactured at lower processing temperature but with the same consolidation force. The nano hardness values in polymer matrix and fibre-matrix interface of CF-PEEK composites manufactured at 950 °C with 350 N consolidation force were about 188.8 and 1297.7 MPa. While these values for composites manufactured at 650 °C using the same consolidation force were about 173.2 and 1077.6 MPa, respectively^25^. Microhardness analysis of unidirectional CF-PEEK specimens manufactured with AFP shows hardness variation between 100 and 151 HV depending on the measurement location^24^.

To date, there have been no investigations on the effects of high-energy ion irradiation on CFRP composites made using advanced manufacturing techniques like AFP^29^. It is well known that about 9% of all cosmic rays arriving at the earth’s atmosphere are α particles^30,7^. For low earth orbits, e.g. at 300 km, the total integral proton fluence over a mission was shown to be about ~ 3.5e^9^ cm^−2^ from 0.1 to 5 MeV and that of solar protons about 1.1e^10^ cm^−2^, from which the total fluence of α particles can be calculated as ~ 3.5e^8^ cm^−2^ and 1.1e^9^ cm^−2^, respectively, assuming α particle fluence is ~ 10% of that of protons. The solar proton intensity is orders of magnitude higher than these during solar events, and since the α particle intensity increases almost at the same rate^31^, it too would be expected to be much higher during times of high solar activity. The studies from Voyager 1 showed that the differential intensity for α particles in the range of 1–10 MeV ranges from ~ 1–3 particles (m^2^ sec sr MeV)^−1^ even in interstellar space^31^. Hence, there is considerable exposure to α particles in space at different distances from the earth.

Thus, the aim of this experimental study is to understand the effects of high energy α particles (He^2+^) on the microstructure and properties of CF-PEEK composites manufactured under different AFP processing conditions. First, the samples were irradiated by α particles (He^2+^). Then, nanoindentation tests were carried out on unirradiated and He^2+^ irradiated samples followed by microstructural analysis on plies' orientations using optical and scanning electron microscopy (SEM). With a good estimate of the range in the radiation induced-damage calculated by the simulation software SRIM, an effort was made to obtain a measure of the change in mechanical properties of the composite due to ion irradiation and to study any influence of fibre orientation on the same.

## Results and discussion

**Figure 1:**
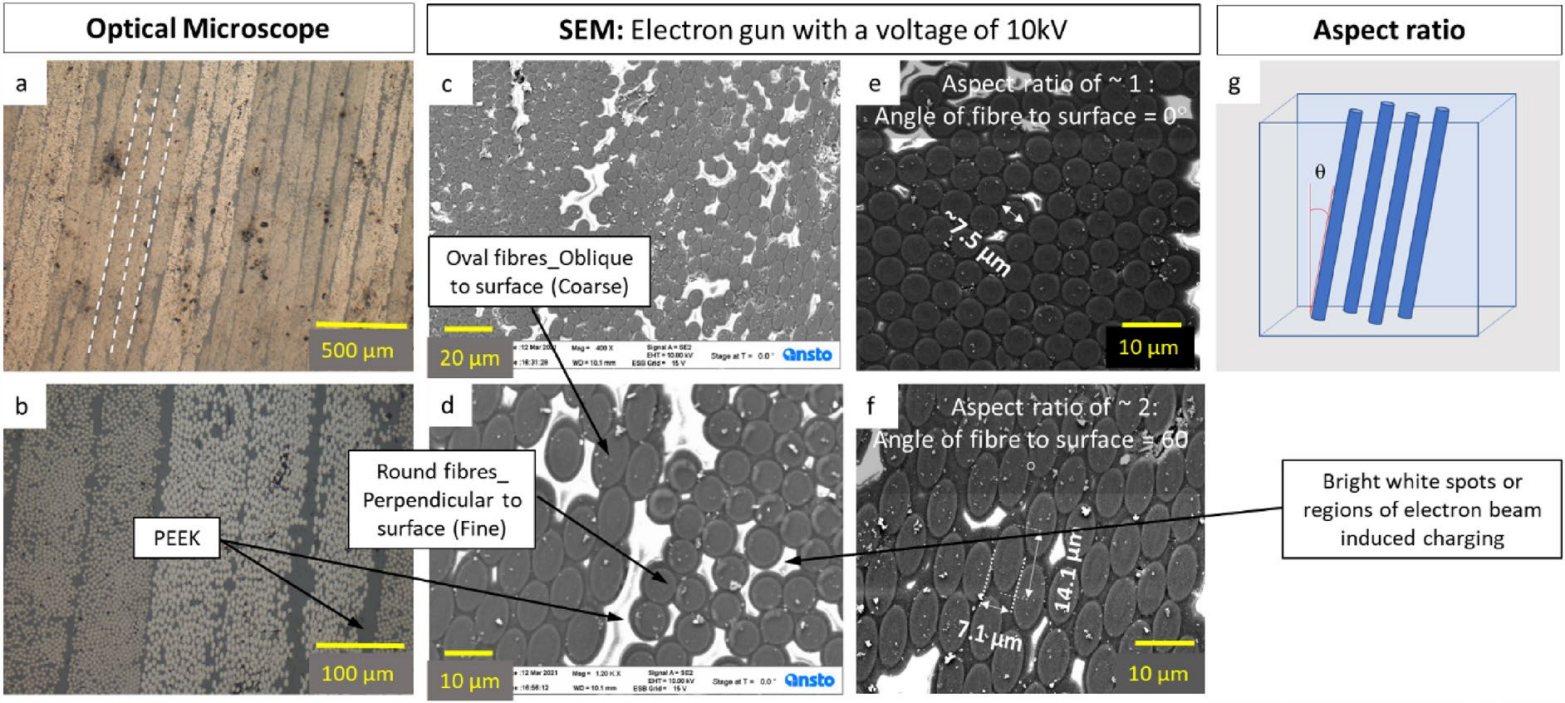
Optical microscope and SEM images for determination of fibre orientation: (**a**) Optical microscopy image of sample “a” at 50 × magnification; (**b**) Optical Image at 200 × magnification to show the circular structures at center; (**c**) SEM image of sample “a” at 400 × magnification; (**d**) Sample “a” at 1200 × magnification; (**e**) Aspect ratio of ~ 1 means angle of fibre to surface = 90°, denoted as “Fine” structure in the text; (**f**) Aspect ratio of ~ 2 means angle of fibre to surface = 60°, denoted as “Coarse” structure in the text and (**g**) calculation of aspect ratio.

The optical microscope (OM) images in Fig. 1a and b show that the carbon fibre composite layers were laid out on top of one another. From OM images it was observed that the indents are more visible when 25 gf are applied. With the application of 5 gf, the materials tend to recover the holes generated by the indenter with time. SEM analysis also provides information on the diameter, shape and orientation of the fibres which helped in the determination of aspect ratios and fibre density across different regions with varying hardness within the material, Fig. 1c and d. Moreover, in certain regions the fibres seemed to exhibit a certain core–shell structure as shown in Fig. 1d. This may be an inadvertent result of the polishing process, as the wear rate of PEEK material is greater in comparison to the embedded carbon fibres. The bright white regions that are observed in the sample during the SEM investigation are the regions of continuous electron accumulation i.e., the regions are seen to be charging up, and highlight the presence of epoxy in the mixture, Fig. 1c and d. Since the PEEK is a non-conductor of electricity, the electrons from the beam get accumulated where there is a large contiguous area of PEEK, and since the imaging is done with the secondary electron detector, areas of greater electron accumulation appear to be brighter, and the brightness is proportional to the number of electrons detected from the same region.

**Figure 2:**
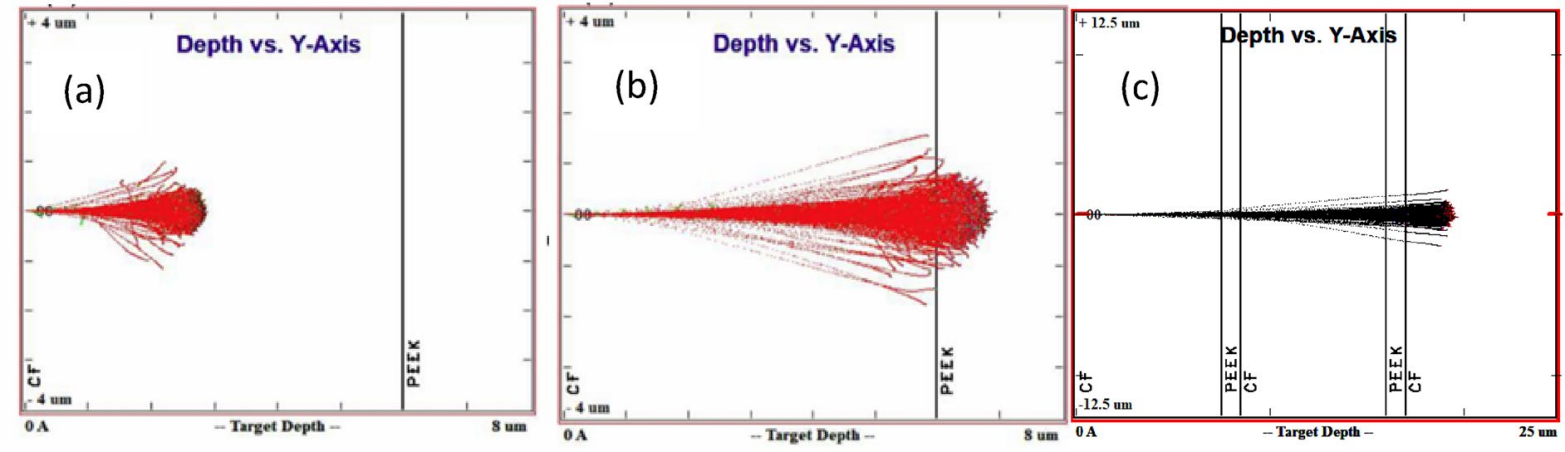
He^2+^ ion irradiation and SRIM calculations: (**a**) Ion energy 1 MeV; (**b**) 2.25 MeV; (**c**) 5 MeV.

The SRIM results showed that α-particles (He^2+^) penetrated to different regions of the modelled composite material (CF-PEEK) on varying the incoming ion energy, as shown in Fig. 2.

Generally, He^2+^ irradiation can lead to material hardening through a combination of physical processes that involve interactions between the ions and the material's atomic structure. Some of the primary mechanisms contributing to material hardening due to He^2+^ irradiation are: displacement damage leading to interstitial and vacancy cluster formation, defect interaction, helium bubble formation, and hardening due to radiation-induced segregation. To elaborate on the last mechanism, helium ions can induce segregation of certain elements to defect sites, leading to changes in the local chemistry.

All these defects produce obstacles for the movement of the shear front (in the case of crystalline materials these are often dislocations), resulting in hardening. Since PEEK, which is the matrix of this composite, is partially crystalline, and the carbon fibres are fully crystalline, it is reasonable to think of the hardening mechanisms applicable in this case to be at least partially analogous to that of metals. Thus, the radiation induced defects are expected to hinder the motion of dislocations and other deformation propagating mechanisms and thus result in hardening.

In order to attain a sufficient depth of irradiated material which will affect both the carbon fibres and the PEEK matrix, and which will, at the same time, allow reliable testing through nanoindentation, a high energy ion beam is desired. This is so because the higher the energy of a beam, the greater is the depth of penetration. This fact was also utilized to produce a more uniform damage layer by introducing a rotating energy-degrader wheel consisting of Al foils of different thicknesses in front of the ion beam. As each foil comes in front of the beam during the wheel’s rotation, it reduces the energy of the outgoing beam by an amount commensurate with its thickness. The thicker the foil, the greater the energy loss, and the lower the energy of the outgoing beam. Thus, when these beams with different energies impinge on the target, they penetrate the surface to a depth which scales with their energy, creating multiple damage peaks at different depths. This has the overall effect of creating a damage layer more uniform than that produced by a single energy beam. This will be illustrated through the use of SRIM simulations in the next paragraph.

The total number of defects and vacancies in the material due to all beam energies were also calculated using SRIM to estimate the damage caused by He^2+^ ion-irradiation. The reason for calculating the damage in terms of defects and vacancies is that this gives a direct methodology for calculating the standard measure of radiation damage, for example, displacements per atom (dpa). This measure provides an internationally recognized standard for assessing radiation damage, which can be compared across different total fluences, fluxes, target materials and energies, as it has been shown to be the measure whose effect on material properties is almost path independent. In other words, that dpa gives a measure of damage, which when the final state is achieved, would have practically the same result on mechanical, electrical or other properties for the same material, irrespective of how this value is arrived at. In crystalline materials, it has been shown in a vast number of cases that there is a direct correlation between damage in terms of dpa and material hardening. The dpa value can be calculated at any depth using Eq. (1):

1 $$dpa = \frac{{n_{v} f.10^{8} }}{\rho }$$

where *n*_*v*_ is the number of vacancies per Å/ion, *f* is the total fluence at a given energy in ions/cm^2^ and *ρ* is the atomic density in atoms/cm^3^.

**Figure 3:**
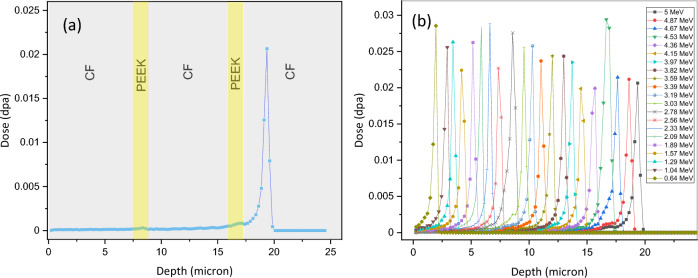
(**a**) Displacement damage dose (in dpa) due to single He ion energy of 5 MeV; (**b**) Displacement damage dose (in dpa) due to multiple energies after ion beam passes through degrader wheel.

The simulated displacement damage dose (in displacements per atom or dpa) due to single He^2+^ ion energy of 5 MeV and the doses (dpa) due to multiple energies after the ion beam passes through the degrader wheel are shown in Fig. 3a and b, respectively. It is apparent from a closer examination of the plots for the individual beams with different energies in Fig. 3b that the higher the beam energy, the greater is the depth of penetration and deeper is the peak damage region. For example, the 5 MeV beam is shown to have a peak at ~ 19.5 µm, while lower energy beams penetrate to shallower depths, culminating in the 0.64 MeV beam with a peak at ~ 2 µm.

It is assumed here that the total fluence is equally distributed at each energy level, since the degrader wheel is divided into 20 sectors with Al foils and an empty sector (in total 21 sectors) all of equal angles. Since the wheel was rotated at a fixed speed, the time spent by each sector in front of the beam was equal. Therefore, the contribution of each foil towards the damage is 1/21th of the calculated damage for the total fluence. The plot in Fig. 3a shows the damage due to (1/21) × 1e^17^ ions/cm^2^ of He ions, and so does each individual curve in Fig. 3b. The sample was simulated as a multilayer composite of alternating carbon fibres of 7.5 µm thickness and PEEK layer of 1 µm thickness. Here it is assumed that the beam is passing through the thickest part or diameter of the fibre and an average thickness of intervening PEEK matrix.

The target is modelled as a composite made of three layers of 8 µm thick carbon fibre alternating with two layers of 1µm thick PEEK. This was done to simulate the situation where the beam is passing through maximum thickness of carbon fibres (across the diameter). Although this is not always the case, it gives an idea of one extreme of the situation and is fairly representative of the overall situation as SRIM calculation showed that there is not much difference in damage between the carbon fibre and PEEK matrix. This is likely due to the abundance of carbon fibre in both cases and the fact that PEEK has other light elements replacing some of the carbon fibre.

**Figure 4:**
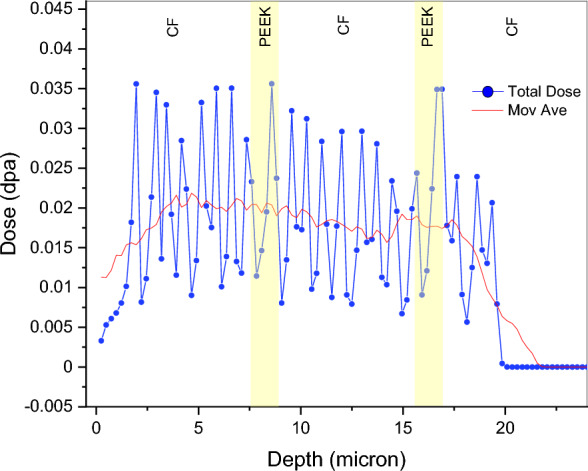
Total displacement damage dose passed through energy degrader wheel, (The red profile shows moving average).

The simulated total displacement damage curve for 5 MeV He^2+^ ions passed through energy degrader wheel for the first five layers of the composite, consisting of alternating carbon fibre and PEEK material, shows a more even distribution than the damage dose of a single energy. As shown in Fig. 4, the average dose is about 0.022 dpa (calculated with E_d_ for carbon = 69 eV), while it would be about ~ 2.8 times that value if the lower value of E_d_ (28 eV) were to be used, according to SRIM. This would result in the average dose to be ~ 0.06 dpa, which is still much less than that in conventional structural materials at similar fluence. For instance, pure Fe would get about 0.11 dpa with the same fluence, MA957 steel would get about 0.24 dpa^32^ while SA508 would receive about 0.1 dpa dose^33^. (These numbers are arrived at by adjusting for the higher fluence in those papers). These differences between the CF-PEEK fibre composite and steels are mainly due to the higher mass density and lower atomic density of these materials, as well as the different values of displacement energy E_d_.

**Figure 5:**
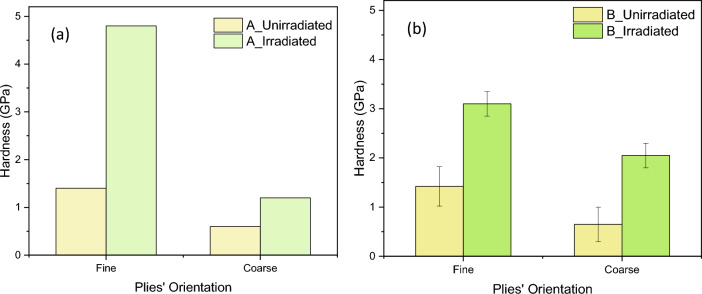
Comparison between the hardness of irradiated and unirradiated samples.

The plots in Fig. 5a and b show the hardness of the unirradiated and irradiated samples A and B, respectively. These data are divided into “Fine” and “Coarse”, which refer to the appearance of the fibres in the top surface section. As indicated in the caption for Fig. 1, in the places where the fibres are perpendicular to the surface, the appearance is fine and, in those places, where the fibres are at oblique angles, the appearance is coarse.

**Figure 6:**
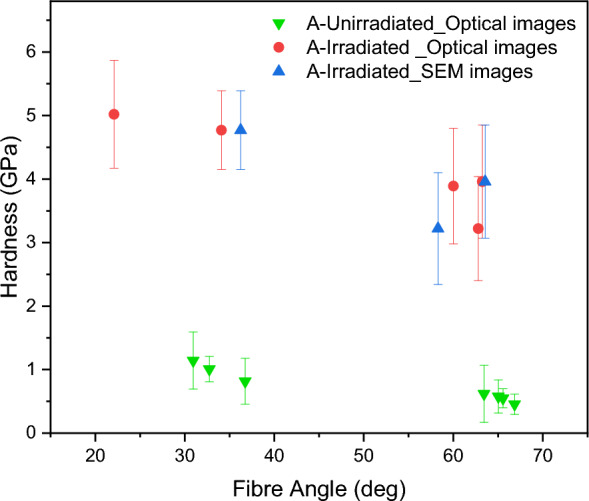
Comparison between the hardness and fibre orientations in sample A.

The effect of fibre orientation on the hardness of unirradiated and irradiated samples is revealed in Fig. 5. Here, it is apparent that for both samples “A” and “B” the average hardness in “Fine” areas, where the fibres are perpendicular to the cross section of the specimens, is higher than that in “Coarse” areas, where the fibres have oblique orientations (Fig. 5). Also, the average hardness values of irradiated samples in both “fine” and “coarse” areas are higher than those in unirradiated samples. The “fine” area in sample “A” shows the greatest irradiation hardening while the “coarse” area shows least absolute hardening. Further detailed analysis was conducted on unirradiated and irradiated specimens of sample A to distinguish between different orientations and fibre packing densities in “Fine” and “Coarse” appearance, as shown in Fig. 6. The exact regions where the indents were made were identified in sample A, through a combination of location coordinates from the nano indenter and the SEM, and the recorded appearance of the indent locations. Thus, the aspect ratios of the fibres in those respective regions were measured. This enabled the determination of fibre orientation precisely using Eq. (2), and as shown in Fig. 6, the hardness decreased with increasing fibre inclination from the indentation axis for both unirradiated and irradiated conditions. This is in complete agreement with the plot of hardness in Sample A as shown in Fig. 5. It is also clear that this variation of hardness with fibre angle is small compared with that caused by irradiation. However, for Sample B, it was more difficult to determine the exact position of the indents, as their marks seem to have collapsed and become obscured or the fibres were broken due to the indentation process. Therefore, it was not possible to carry out the aspect ratio analysis on Sample B, though the “Fine” and “Coarse” region observations point strongly to a very similar behaviour to that of Sample A.

## Methods

### Sample preparation

**Figure 7:**
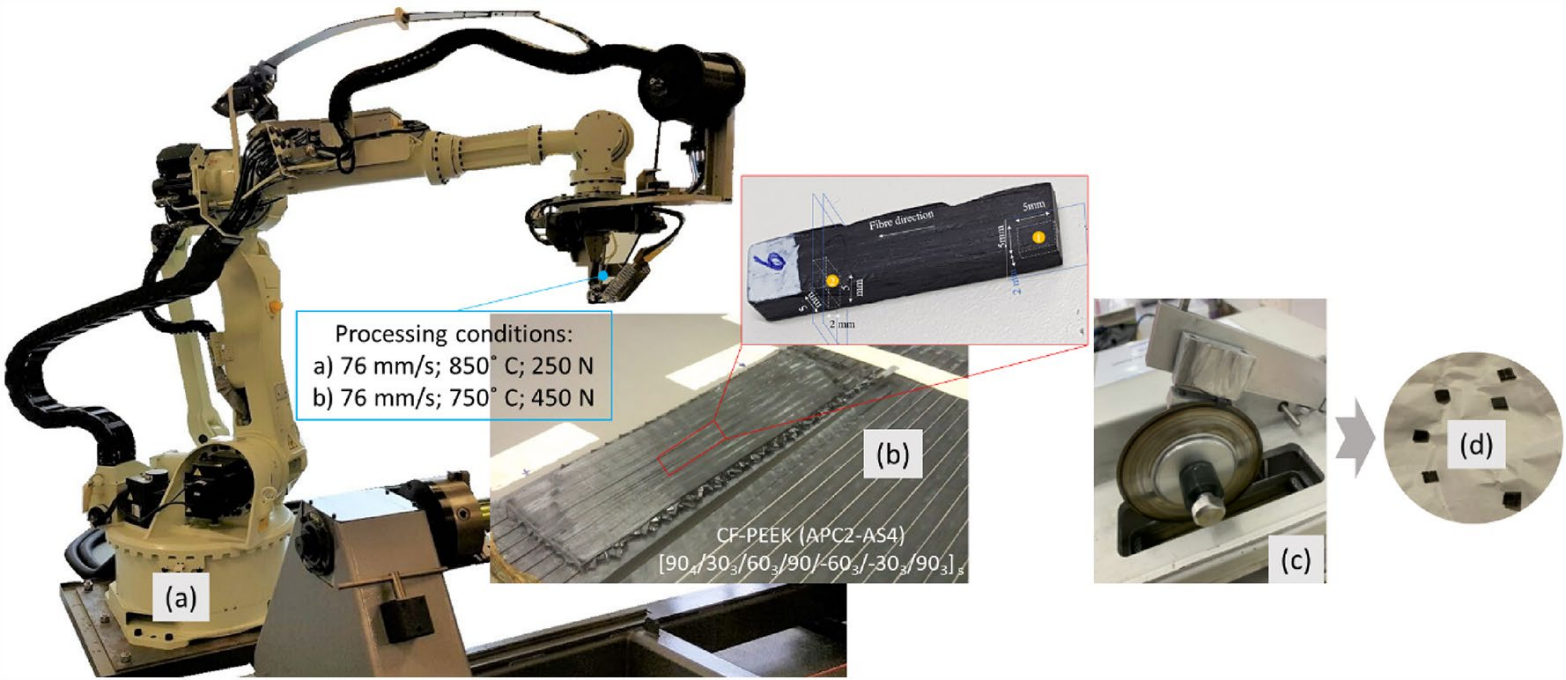
(**a**) Automated fibre placement machine with thermoplastic head; (**b**) Test Samples; (**c**) Low Speed Cutter; (**d**) Coupon samples.

In this experimental study, two composite panels, each consisting of forty plies of unidirectional thermoplastic CF-PEEK prepreg tape (AS4-APC2; supplied by Solvay®) were manufactured using a Trelleborg Sealing Solutions ® (ex. Automated Dynamics) built AFP. The overall dimensions of panels were 160 mm × 60 mm × 6 mm with stack-up sequence of [90_4_/30_3_/60_3_/90/–60_3_/–30_3_/90_3_] _s._ The prepreg tapes used for making the panels were 6.35 mm wide with a thickness of 0.15 mm. The material properties of CF-PEEK are summarized in the Table 1. During the lay-up process the prepreg plies were treated by a heating and cooling cycle. The panels were manufactured using two different processing conditions in which the deposition rate was kept constant at 76 mm/s while the temperature and consolidation force were varied. (A: HGT temperature = 850 °C, consolidation force = 250 N; B: HGT temperature = 750 °C, consolidation force = 450 N)^23^. Following that, the panels were cut to 5 mm × 5 mm × 2 mm coupons using Low Speed Cutting machine (150 rpm) as shown in Fig. 7. The cut samples then proceeded for polishing and grinding process using Struers® Labo-Pol 60™ machine. The top and cross-sectional surfaces of the cut samples were polished and ground initially using a 320–4000 grit size silicon carbide (SiC) paper/colloidal silica. The final surface finish of polished samples was < 1 μm.

**Table 1:** Mechanical properties of CF-PEEK.

Density	Elastic modulus	Shear modulus	Poisson’s ratio	Fibre volume fraction
1570 kg/m^3^	138 GPa	5 GPa	0.28	0.6

### Ion-irradiation

The STAR accelerator at ANSTO was used to irradiate the material with α particles (He^2+^). The Stopping and Range of Ions in Matter (SRIM) and Transport of Ions in Matter (TRIM) software was used to estimate the extent of penetration of α-particles (He^2+^) and the resultant displacement damage in the different regions of samples^34^. SRIM is a quantum mechanics-based Monte-Carlo simulation program that allows the calculation of the penetration depth of ions and the damage caused by them by treating the target as amorphous and all collisions as binary interactions. It uses the displacement energy of atoms in the structure to determine whether an atom will be displaced from its position on being impinged by an incoming ion or a knock-on atom.

Since the SRIM software assumes that the target is made of atoms with an average neighbour distance given by the material density, but with no particular ordering, i.e. it is amorphous, the displacement energies used are appropriately the average displacement energies for all directions.

The calculation of damage and stopping is rendered extremely efficient by using statistical methods which treat the ion as making jumps between the calculated collisions and then average the collisional results over the intermediate gap^34^.

The displacement energy (E_v_) of the various types of atoms was specified as follows: C—69eV^35^, H—10 eV and O—28 eV. There is some uncertainty in the use of these values, as they vary for each element from compound to compound depending on the chemical bonding. Calculations using E_v_ values of C ranging from 28 eV (default value in SRIM) to 69eV (as per Konobeyev et al.^35^), for instance, have shown the vacancies per ion to vary from 118 to 77 in PEEK (35% reduction), and 89 to 32 in pure C (65% reduction). Thus, if these are considered to be the minimum and maximum values of E_v_ for C, one may assume a reduction of up to about 35–65% in the damage dose at the higher E_d_ values. The high value of E_d_ used in this study gives a lower estimate of the damage, which can be multiplied by a correction factor based on the SRIM simulations to estimate the upper limit of damage.

**Figure 8:**
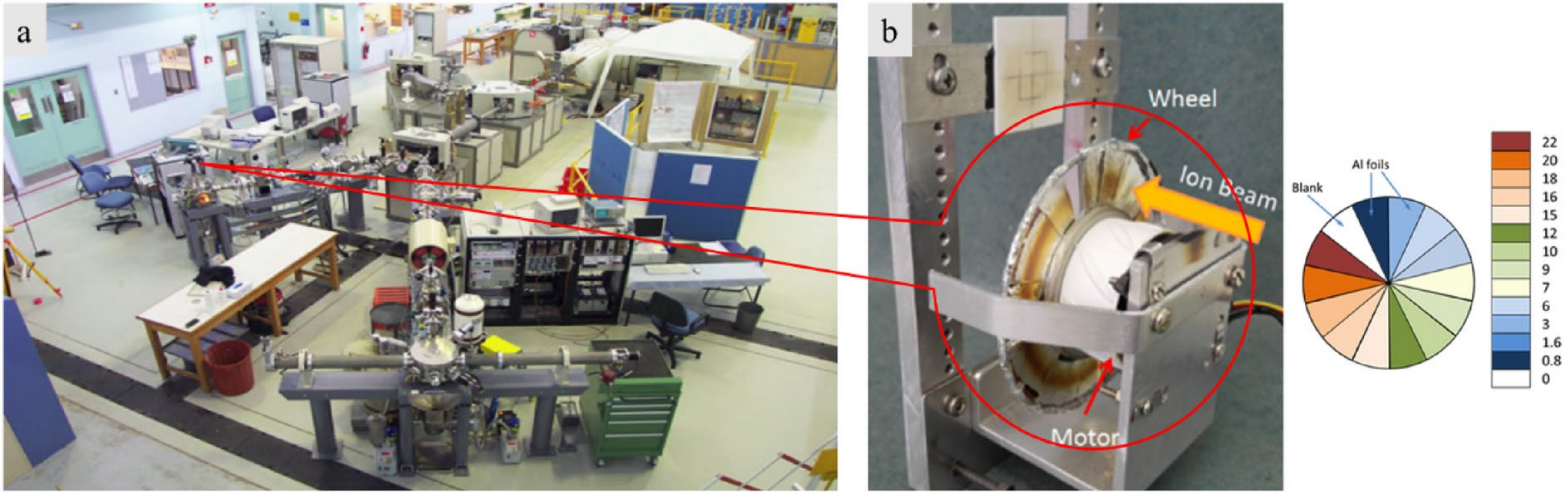
(**a**) 2MV STAR particle accelerator at ANSTO; (**b**) A typical degrader wheel showing sectors of Al foil with different thicknesses in microns. The actual one used in this case had 20 sectors of Al foils with equal angles.

The SRIM simulations showed that the radiation damage layer is shallow (up to ~ 20 µm for 5 MeV He ions and 38 µm for 2 MeV protons). As expected, the depth of the damage does not depend on the displacement energy, but only on the incoming ion energy. In this experimental study, the polished samples were irradiated with a beam energy of 5 MeV He^2+^ ions to a total fluence of 1e^17^ ions/cm^2^ using an energy degrader wheel in the STAR accelerator Fig. 8. The degrader wheel consisted of twenty sectors made of Al foils of increasing thickness, which led to the attenuation of the beam energy to varying degrees, thus resulting in a more uniform damage through the depth of the ion range.

While there is obviously a difference between the assumption of SRIM that the structure is amorphous, and the reality that it may be partially or fully crystalline, it has been shown in multiple experimental studies that the depth of penetration is indeed very close to that predicted by SRIM, and that the extent of damage is proportional to the damage calculated by SRIM^36–39^.

### Image acquisition

Structural analysis of prepreg tape were carried out using a Zeiss® Optical Microscope (OM) and Zeiss® UltraPlus™ Scanning Electron Microscope (SEM). The SEM was run at a voltage of 10 kV and the secondary electron detector was used to image the sample, which was painted on the side with C paste to make it conductive for SEM characterization. SEM analysis provided information on the diameter, shape and aspect ratios of the fibres which subsequently helped in the determination of orientation across the regions of varying hardness within the material using Eqs. (2) and (3):

2 $$Aspect _{ratio} = \frac{Major\;axis}{{Minor\;axis}}$$

3 $$\cos \theta = \frac{1}{Aspect\;ratio}$$

### Nanoindentation

Nanoindentation analysis was carried out using an Agilent® / MTS® G200™ available at the NMDC research facility at ANSTO. The nanoindentation was carried out in the load-controlled mode with a Berkovich diamond tip at maximum loads of 50 mN and 250 mN and along linear arrays parallel to the traces of the fibre plies in the cross-section of the composite blocks. For the correlation of the indents with the measured hardness values at the respective sites, the location of the indents with respect to the sample corners was noted from the nano indenter setup, which used an inverted XY coordinate system. For additional ease of location, larger reference markers in an “*H*” pattern were used. The hardness of the material was determined using Eq. (4)^25^,

4 $$H = \frac{{P_{max} }}{{A_{c} }}$$

where *P*_*max*_ is peak loading force achieved and *A*_*c*_ is projected contact area. As the indenter tip had a triangular pyramid structure, the area of cross-section is related to the indentation depth by Eq. (5),

5 $$A_{c} = 25.4d^{2}$$

where *d* is indentation depth and 25.4 is the area function for a Berkovich indenter with tip angle approximately 65.27°^40^.

## Conclusions

In this experimental study, the effect of high energy He^2+^ particles on the microstructure and properties of CF-PEEK composites manufactured using AFP under two different processing conditions was investigated. Microstructural analysis using optical microscope and SEM was carried out on unirradiated and irradiated samples followed by nanoindentation testing. Further optical and SEM characterization was performed after the nanoindentation testing to understand the effects of fibre orientation on hardness before and after irradiation. Some of the conclusions from these tests are as follows:

(i) The measured hardness of the composite depends on the orientation of fibres, and the orientation perpendicular to the surface is the hardest, while it decreases with increasing fibre inclination from the surface normal.
(ii) SRIM simulations were used to estimate the damage caused by 5 MeV He ions using a degrader wheel in a composite modelled by 3 layers of 7.5 µm carbon fibres alternating with 2 layers of 1 µm thick PEEK. It was found that the average damage dose for a total fluence of 1 × 10^17^ ions /cm^2^ was about 0.022 dpa (for E_d_ = 69 eV) and ~ 0.06 dpa (for E_d_ = 28 eV) through a depth of ~ 20 µm and there was not much difference in damage between the carbon fibre and PEEK.
(iii) Irradiation by He ions causes hardening of the CF-PEEK composite, ranging from 0.7 GPa to 3.5 GPa, depending on the processing condition and fibre orientation. This level of hardening is very high with respect to the low dose when it is compared with the hardening observed in metals.
(iv) Fibre orientation affects the hardness in a statistically significant manner—there is ~ 0.5–1 GPa decrease in hardness when the inclination of the fibres increases from ~ 30° to ~ 60° for both samples “A” and “B”, in both unirradiated and irradiated condition.
(v) The initial average hardness of unirradiated sample “A” was ~ 0.6–0.8 GPa, while that of sample “B” was ~ 1 GPa.
(vi) Irradiation increases the hardness of the fibres in all cases. For sample “A”, the increase is about 3–4 GPa, while for sample “B”, it is about 1.3–1.6 GPa.
(vii) Although the unirradiated hardness of sample “B” was slightly higher, the increase in hardness after irradiation was appreciably lower in sample “B” than in sample “A”.

## Data Availability

The data that support the findings of this study are available from the corresponding author upon reasonable request.
